# Nitrous oxide-induced neurotoxicity: Clinical characteristics and impacts on overall neurological impairments

**DOI:** 10.3389/fneur.2023.1132542

**Published:** 2023-02-23

**Authors:** Hui Gao, Ruihan Wang, Yan Zeng, Linyuan Qin, Hanlin Cai, Dong Zhou, Qin Chen

**Affiliations:** ^1^Department of Neurology, West China Hospital, Sichuan University, Chengdu, Sichuan, China; ^2^Neurobiological Laboratory, West China Hospital, Sichuan University, Chengdu, Sichuan, China

**Keywords:** nitrous oxide (N_2_O), neurological impairments, neurotoxicity, management, clinical characteristics

## Abstract

**Objective:**

The reports of the recreational use of nitrous oxide (N_2_O) and its related neuropathy are increasing. However, it is unclear whether specific clinical characteristics are associated with the overall neurological impairments among these individuals.

**Methods:**

We retrospectively included 20 hospitalized patients with N_2_O-related neurological complaints between January 2016 and March 2021 at the West China Hospital of Sichuan University. Detailed demographic, clinical features, lab tests, and imaging data were collected. A functional disability rating score (FDRS) was calculated to determine the degree of neurological impairment. The relationships between the aforementioned factors and the FDRS sum score were explored.

**Results:**

These individuals were aged between 16 and 30 years (mean ± SD: 21.90 ± 4.06). At admission, unsteady gait (95%, nineteen of twenty), weakness (95%, nineteen of twenty), and limb paresthesia (70%, fourteen of twenty) were the most common symptoms; decreased deep tendon reflexes (100%, nineteen of nineteen), reduced muscle strength (95%, nineteen of twenty), and impaired coordination (95%, nineteen of twenty) were frequently found. The FDRS sum scores ranged from 3 to 12. Among all the factors, admission from the emergency room (*p* = 0.033), decreased hemoglobin (*p* = 0.004) (without previous VitB12 supplements), decreased red blood cell (RBC) count (*p* = 0.004) (without previous VitB12 supplements), and increased mean corpuscular volume (*p* = 0.036) (with previous VitB12 supplements) positively correlated with the FDRS sum score.

**Conclusion:**

Nitrous oxide (N_2_O) could lead to severe neurological impairments among users. Abnormal RBC indicators at admission may be associated with a worse clinical presentation and need further attention. Population education about the consequences of N_2_O consumption and control measures concerning access to N_2_O should be further emphasized.

## Introduction

Traditionally, nitrous oxide (N_2_O) was widely used in anesthetic practices (1). With the propensity to induce euphoria and simple accessibility, the recreational use of N_2_O has expanded and become popular (2).

However, prolonged exposure to N_2_O could have consequences. In 1978, neuropathy caused by N_2_O abuse was first reported in three cases, followed by descriptions of 12 additional cases of abuse or occupational exposure (3, 4). It is widely believed that N_2_O could oxidize the cobalt ion of Vitamin B12 (VitB12), disrupt the function of cobalamin, and cause a disturbance in myelin sheath synthesis and maintenance (5). Demyelination of white matter in the brain, the dorsal and lateral spinal cord, and the peripheral nerves can later lead to gait disturbances, paresthesia, autonomic dysfunction, and cognitive impairment.

Due to a lack of supervision measures and a large population, the number of cases of N_2_O-related neurological disorders in China has increased rapidly since 2016 (6–12). Published studies have typically focused on symptom presentations, imaging characteristics, laboratory findings, and distinctions from other subacute combined degenerations (SCDs). The relationship between symptoms and the presentation of clinical severity has been understudied and N_2_O abuse is still an overlooked etiology for SCD or polyneuropathy among young patients.

Thus, we conducted this retrospective observational study with detailed data collected from multiple cases in a single center to investigate the characteristics of N_2_O consumption history, clinical features, as well as medical examination results, and their effects on the overall neurological impairments of these individuals.

## Methods and materials

### Participants and baseline evaluation

We retrospectively reviewed and analyzed the records of patients who reported N_2_O use, presented with neurological symptoms, and were admitted to the Department of Neurology at the West China Hospital of Sichuan University between January 2016 and March 2021. Data collected included demographic, clinical, laboratory, neuroimaging, and electromyography (EMG) from the first N_2_O-related hospitalization record. After thoroughly evaluating the aforementioned data, two neurologists confirmed the final diagnosis of N_2_O-related neurological disorders. According to the Helsinki Declaration, the Ethics Committee of the West China Hospital of Sichuan University approved this study. All written consents were collected from the participants or their legal guardians.

### Demographic and clinical assessment

Demographics, including sex, age of onset, occupation, past neuropsychological and other substance consumption history, history of N_2_O use, onset-to-admission time, ways of admission to the hospital, length of hospital stay, abnormal events during hospitalization, recovery status at discharge, and records of natural follow-up at our outpatient clinic were collected. The history of N_2_O use consists of the length of exposure, the recent increase in use, and the concealment of N_2_O consumption at the first admission.

Neurological symptoms and signs were comprehensively assessed during hospitalization. Based on the clinical evaluations, the functional disability rating score (FDRS) was calculated by two experienced neurologists. The score has been used widely in the clinical severity evaluation of SCDs, including N_2_O-related neuropathies (11, 13, 14). The five-part scoring system is described as follows: (1) gait (0 = normal, 1 = positive Romberg's sign, 2 = impaired but able to walk unsupported, 3 = substantial support required for ambulation, 4 = wheelchair-bound or bedridden); (2) sensory disturbances including hypesthesia, dysesthesia, vibration/joint-position impairment (0 = normal, 1 = impairment only in toes and fingers, 2 = impairment in the ankles and wrists, 3 = impairment in the upper arms and legs); (3) mental impairment (0 = normal, 1 = intellectual or behavioral impairment requiring no social support, 2 = partial dependence for all activities of daily living, 3 = complete dependence for all activities of daily living); (4) neuropathy (0 = normal reflex, 1 = loss or reduction of deep tendon reflexes of the ankle, 2 = loss or reduction of deep tendon reflexes of the patella, 3 = loss or reduction of deep tendon reflexes of the biceps); and (5) pyramidal tract signs (0 = normal, 1 = positive Babinski sign, 2 = spastic paraparesis, 3 = spastic tetraparesis). The cumulative score ranges from 0 to 16. A higher score indicated increased severity of neurological impairment and a worse functional status of a patient.

### Laboratory, magnetic resonance imaging (MRI), and electromyography (EMG) assessments

We collected the first routine blood results, VitB12-related indicators, and cerebrospinal fluid (CSF) results of the included individuals after admission. Due to the possibility that VitB12 supplements could affect routine blood results and VitB12-related indicators, we classified blood results based on whether the individual had VitB12 supplements before being admitted.

During hospitalization, spinal or head MRI was performed by 1.5T MR devices (Magnetom H-15 and Vision; Siemens, Erlangen, Germany). Depending on the clinical evaluations, the spinal cord segments and the head were scanned with sagittal and axial reconstruction. The scanning sequences included T1-weighted imaging (T1WI), T2-weighted imaging (T2WI), and fluid-attenuated inversion recovery (FLAIR) sequences. T1WI (repetition time/echo time: 500–550/10–15) and T2WI (repetition time/echo time: 3,000–4,000/100–120) were performed with echo train lengths of 5. Other MRI scan parameters included a 3-mm section thickness and a 1-mm scanning interval. The locations and signs of the MRI abnormalities were recorded and collected.

Electromyography (EMG) of the median, ulnar, peroneal, tibial, and sural nerves was available for most of the patients. The compound muscle action potential (CMAP) amplitude, distal latency, conduction velocity, and the amplitude and conduction velocity of the sensory nerve action potential (SNAP) were measured. Based on the EMGs, an experienced neurologist, and an experienced EMG technician further classified the individuals as (1) sensorimotor, motor, or sensory neuropathy; and (2) axonal damage dominant type or demyelination dominant type.

### Statistical analysis

Data with normal distribution were presented as means and standard deviations (SD) (x ± s), and data with non-normal distribution were presented as the medians and interquartile range (IQR, 25th and 75th percentiles). Qualitative data were described as counts and percentages. Pearson (qualitative data with normal distribution) or Spearman correlation analysis was performed to examine the correlations between the demographics, lab tests, MRI variables, and the FDRS sum score. A value of *p* < 0.05 was considered statistically significant. The analysis was performed using SPSS 28.0 (SPSS Inc., Chicago, IL, United States).

## Results

### Demographics

The study enrolled 20 (male/female: five of fifteen) patients aged between 16 and 30 (mean ± SD: 21.90 ± 4.06) with N_2_O-related neurological disorders (Table 1). The time from symptom onset to admission days (median: 14.5 days, IQR: 9–120 days) was much shorter than the exposure time of N_2_O (median: 7 months, IQR: 4–24 months). Seventy-five percent (nine of twelve) of the individuals reported recent increases in their use of N_2_O, but approximately one-third (30%, six of twenty) of them intentionally concealed their history of N_2_O consumption during admission. The accurate quantification of the consumption amount was difficult because patients could inhale in balloons/bulbs or canisters, and they often inhaled with a group of people.

**Table 1:** Demographics, history of N_2_O use, and hospitalization of the patients.

**Demographics**	
Male: Female	5:15
Age (years)	21.90 ± 4.06
Unemployed (%)^a^	55.00% (11/20)
Positive past neuropsychological history (%)^b^	25.00% (5/20)
Positive substance consumption history (%)^c^	55.00% (11/20)
**History of N**_2_ **O use**	
N_2_O exposure time (months)^d^	7.00 (IQR:4.00–24.00)
Recent increased use (%)^d^	75.00% (9/12)
Concealment of the use (%)	30.00% (6/20)
**Hospitalization**	
Onset-to-admission times (days)	14.50 (IQR:9.00–120.00)
Admitted from Emergency Room (%)	45.00% (9/20)
Length of hospital stay (days)	9.20 ± 3.65
Abnormalities during hospitalization	50.00% (10/20)
symptom worsening^e^	20.00% (4/20)
severe negative emotion^f^	40.00% (8/20)
Improvement at discharge	75.00% (15/20)
sensory symptoms^d^	71.43% (10/14)
motor symptoms	65.00% (13/20)
Natural follow-up rate (%)^g^	50.00% (10/20)

Data with normal distribution were presented as means and standard deviations (SD) (x ± s); data with non-normal distribution were presented as the medians and interquartile ranges (IQRs). Qualitative data were described as counts or percentages.

a Define unemployed as currently without a job or being a student.

b Positive past neuropsychological history includes a diagnosis of psychiatric disorder or sleep disorder.

c Substance consumption includes narcotics, nicotine/cigarette smoking, and alcohol.

d Include individuals with histories available for the analysis.

e Previous symptoms/signs got worse or new symptoms/signs appeared.

f During hospitalization, patients are routinely assessed for their emotional wellbeing using the Huaxi emotional index (HEI), a nine-question-based scale that has been validated to screen for depression and anxiety. The answer to each item ranged from 0 to 4 (sum up to 36), representing from “none” to “all the time.” The scale defines a severe negative emotion as an HEI≥17.

g Include natural follow-up after hospitalization in our outpatient neurology clinic.

More than half of the participants (55%, eleven of twenty) were unemployed or had a history of other substance consumption (narcotics, nicotine/cigarette smoking, and alcohol). A quarter of the included individuals (five of twenty) had positive past neuropsychological history, including depression, anxiety, or sleep disorder.

### Clinical characteristics

The most common clinical symptoms on admission were unsteady gait (95%, nineteen of twenty), weakness (95%, nineteen of twenty), and limb paresthesia (70%, fourteen of twenty). Paresthesia and weakness were more prevalent in the lower extremities than in the upper extremities. There were presentations other than sensory or motor symptoms. Six out of 20 individuals showed autonomic nervous dysfunction like constipation or sweatiness. Few had cognitive impairment (15%, three of twenty), hallucination (10%, two of twenty), seizure (5%, one of twenty), dystonia (5%, one of twenty), or urinary/fecal incontinence (5%, one of twenty).

There were several frequent signs observed during the neurological examination, including decreased deep tendon reflexes (100%, nineteen of nineteen), decreased muscle strength (95%, nineteen of twenty), and impaired coordination (95%, nineteen of twenty). The abnormal deep tendon reflexes were mainly found in the biceps (45%, nine of twenty) and patella (30%, six of twenty). Eight individuals (40%, eight of twenty) exhibited pathological signs of damage to the pyramidal tracts. Approximately one-third (30%, six of twenty) also had altered mental status.

The sum score of the comprehensive five-part FDRS assessment ranged from 3 to 12 (mean ± SD: 8.10 ± 2.22). Gait (median with IQR: 3, 2.00–3.00) and sensory disturbance (median with IQR: 3, 2.00–3.00) were frequently found and being more severe, followed by neuropathy (changes in reflexes) (median with IQR: 2, 1.25–3.00). While influence on mental function (median with IQR: 0, 0.00–1.00) and pyramidal tract damage (median with IQR: 0, 0.00–1.00) were less frequent and slight.

Severe negative emotion [evaluated by the Huaxi emotional distress index (HEI) (15)] occurred in eight individuals during hospitalization. After hospital stay (mean ± SD: 9.20 ± 3.65), although 75% (fifteen of twenty) of individuals had improvement at discharge, only half of them had follow-up visits at our outpatient neurology clinic (Table 1).

### MRI and EMG findings

**Figure 1:**
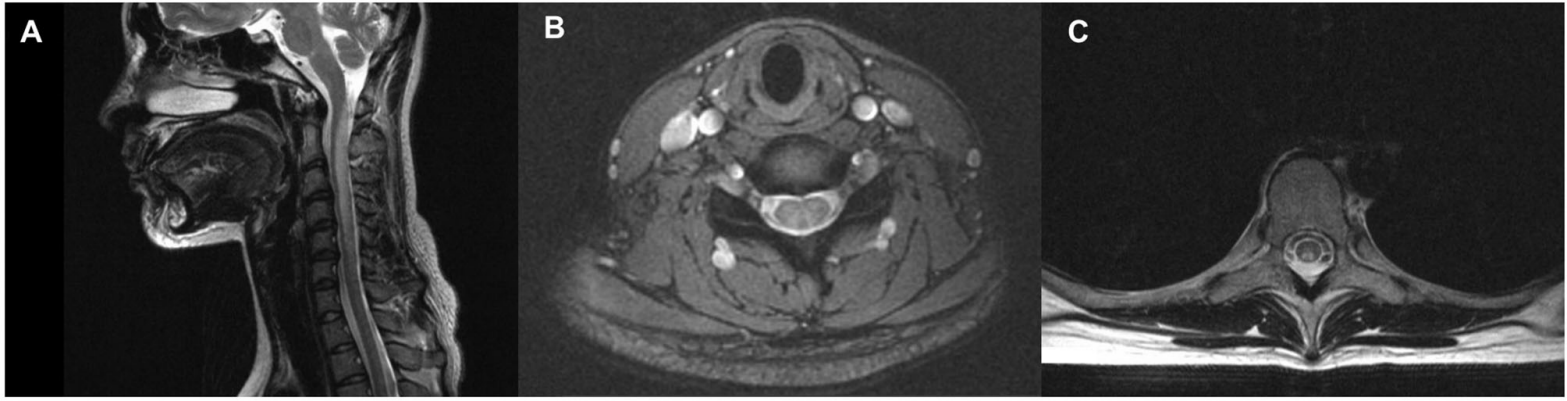
Spinal cord Magnetic Resonance Imaging (MRI) of a patient with N_2_O -abuse who complained of progressive unsteadiness while walking and limb numbness for 14 days. **(A)** Sagittal T2 MRI of the cervical spine showed a longitudinal hyperintensity signal C1–C6. **(B)** Axial gadolinium-enhanced T2 MRI showed symmetric and inverted V-shaped hyperintense signals in the dorsal columns of the cervical spinal cord. **(C)** Axial gadolinium-enhanced T2 MRI showed symmetric and inverted V-shaped hyperintense signals in the dorsal columns of the thoracic spinal cord.

According to the clinical evaluations during hospitalization, MRIs of the head and any spine segment were performed on 13 and 16 individuals, respectively (Table 2). MRIs of the head revealed unspecific demyelination in two of 13 individuals. Five (33.33%, five of fifteen) had T2-weighted signal intensities in the cervical vertebral levels, and three (20%, three of twenty) had T2-weighted signal intensities in the thoracic vertebral levels. There were large variations in the lesion size; however, the lesion mostly extended for four or more segments (Table 2). The inverted “V” sign was observed in the cervical/thoracic vertebral levels of four patients (Figure 1).

**Table 2:** Magnetic resonance imaging (MRI) findings of the patients.

	**Percentages of abnormality (%)**	**Description of lesion**
Head MRI (*n* = 13)	15.38% (2/13)	Unspecific demyelination
Spine MRI (*n* = 16)	37.50% (6/16)	Lesion size of the 6 patients by vertebral level^a^: C4–C5 (inverse V) C2–C5 (inverse V) C1–C7 (inverse V), T6-T11 (inverse V) C2–C6 C1–C6 (inverse V), T5-T10 (inverse V) T2
Cervical level (*n* = 15)		
Inverse V sign (%)	26.67% (4/15)	
Other T2-weighted signal intensities (%)	6.67% (1/15)	
Thoracic level (*n* = 15)		
Inverse V sign (%)	13.33% (2/15)	
Other T2-weighted signal intensities (%)	6.67% (1/15)	
Lumbar level (*n* = 6)		
Inverse V sign (%)	0.00% (0/6)	
T2-weighted signal intensities (%)	0.00% (0/6)	

a “C” stands for “Cervical”, T stands for “Thoracic”.

All patients (100%, fifteen of fifteen) had abnormal EMG findings during evaluations. Sensorimotor neuropathies were found in 80.0% (twelve of fifteen) of patients, and the motor nerves were more severely involved. The other three patients showed purely motor neuropathy. Most of the individuals had axonal damage rather than demyelinating, and lower extremities were predominantly affected (Table 3).

**Table 3:** Electromyography (EMG) and lab findings of the patients.

	**Percentages or median**	**Supplementary information^b^**
**EMG**		
Rate of abnormality (%)^a^	100% (15/15)	–
Axonal damage dominated	80% (12/15)	–
Demyelinating dominated	20% (3/15)	–
Motor damage dominated	100% (15/15)	Three had normal ranged sensory nerve conduction study values.
Lower extremities dominated	93.33% (14/15)	
**First blood laboratory results without previous VitB12 supplements (*****n*** = **10)**		
Decreased Hb	40.00% (4/10)	Decrease Hb was defined as <130g/L in male, and <115 g/L in female.
Increased MCV	20.00% (2/10)	Increased MCV was defined as > 100 fL
Decreased RBC count	40.00% (4/10)	Decrease RBC count was defined as < 4.3 * 10^12^/L in male, and < 3.8 * 10^12^/L in female.
VitB12 level	161.000 (IQR:106.000–563.000)	Normal range: 180–914 Pg/ml
VitB12 deficiency	57.14% (4/7)	
Increased Hcy	60.00% (3/5)	Hcy was considered abnormal if ≥15 μmol/L
**First blood lab results with previous VitB12 supplements (*****n*** = **6)**		
Decreased Hb	16.67% (1/6)	
Increased MCV	33.33% (2/6)	
Decreased RBC count	33.33% (2/6)	
VitB12	1,500.000 (IQR:604.750–1515.000)	
VitB12 deficiency	0.00% (0/6)	
Increased Hcy	75.00% (3/4)	

Hb, hemoglobin; MCV, mean corpuscular volume; RBC, red blood cell; Hcy, homocysteine; IQR, interquartile range; VitB12, vitamin 12.

a Include patients with positive EMG findings in our hospital. There was one patient with positive EMG findings in another hospital, but not available with the analysis. Three patients were not able to cooperate during the EMG study.

b These definitions and normal ranges were according to the reference range at the laboratory department of West China Hospital.

### Lab tests

We summarized the changes in serum levels of hemoglobin (Hb), mean corpuscular volume (MCV), red blood cell (RBC), VitB12, and homocysteine of the patients, and then categorized them based on whether they had taken vitamin B12 supplements before admission (Table 3). Forty percent (four of ten) of the individuals without previous VitB12 supplementation and 16.67% (one of six) of the individuals with previous VitB12 supplementation showed decreased Hb levels. Similarly, decreased RBC count and increased MCV could also be found in both groups. More than half of the participants (four of seven, 57.14%) without previous VitB12 supplements were deficient in VitB12; however, participants with previous VitB12 supplements did not have reduced serum VitB12.

### Factors related to the functional status

We examined the relationship between the demographic features, N_2_O consumption, positive MRI findings, abnormal lab results, and the FDRS sum score. Among all of them, admission from the emergency room (Spearman correlation coefficient = 0.477, *p* = 0.033), decreased Hb (Spearman correlation coefficient = 0.817, *p* = 0.004, in individuals without previous VitB12 supplements), and decreased RBC count (Spearman correlation coefficient = 0.817, *p* = 0.004, in individuals without previous VitB12 supplements), and increased MCV (Spearman correlation coefficient = 0.840, *p* = 0.036, in individuals with previous VitB12 supplements) were positively correlated with the FDRS score (Supplementary Table 1).

## Discussion

We conducted this retrospective observational study on 20 individuals with N_2_O-induced neuropathy from a tertiary medical center. We found that clinical characteristics, imaging features, and serum VitB12 levels were not related to the overall clinical severity; however, a decrease in Hb and RBC counts and an increase in MCV were associated with a worse clinical presentation upon admission.

The FDRS score was to quantify the severity of clinical manifestations and the effect on physical activity in SCDs, including N_2_O-related neuropathies (11, 13, 14). There may be objective factors related to the FDRS sum score, such as demographic characteristics, N_2_O consumption, hospitalization characteristics, imaging, and laboratory results. Therefore, these may indicate a worse clinical picture or outcome and provide insight into the mechanism of N_2_O consumption-related neurological damage. We found that decreased Hb and RBC counts were associated with a lower FDRS score on admission in individuals who had or had not previously taken VitB12 supplements. Similarly, a higher MCV was associated with a lower FDRS score on admission in individuals who had previously taken VitB12 supplements. Although these relationships have not been reported in other recreational N_2_O-induced neuropathies, postsurgical high MCV has been shown to be associated with a more severe clinical picture and poorer recovery (tendency) in anesthetic N_2_O-induced neuropathies (16, 17). In other SCDs unrelated to N_2_O, a correlation between a lower Hb level and a more severe clinical presentation has also been identified (18). Thus, changes in the RBC index on admission may be a sensitive indicator of more severe clinical presentation.

The majority of studies, including our own, failed to find a correlation between N_2_O cumulative consumption and the severity of initial clinical presentation or outcome, regardless of different evaluation ways (17, 19–21). There were contradictory findings regarding VitB12 and clinical severity, possibly because different studies included varying proportions of VitB12-supplemented patients before admission (11, 17, 19, 20). Thus, we categorized our patients according to whether they had taken VitB12 supplements before admission. Even after categorizing the data, we were unable to identify any correlations between serum VitB12 level and the FDRS sum score. Meanwhile, studies have found total VitB12 (19, 21) and active VitB12 levels (19) did not correlate with cumulative nitrous oxide use (19). These data suggested that N_2_O-induced neurotoxicity might not be dose-dependent. Among our participants, the mean N_2_O exposure time was 7 months, while the mean symptom onset-to-admission days was only 8.5 days. In addition to this finding, 75% of the patients reported increases in the use of N_2_O prior to admission, suggesting that symptoms could be caused by predisposing subclinical functional declines of VitB12 and a rapid process of cobalamin inactivation. Similar conditions were observed in N_2_O-anesthetized patients with postoperative neuropathy, and their N_2_O exposure was relatively rapid. Studies have found that some of them had presurgical recreational N_2_O use caused MCV increase but were asymptomatic before surgery (16, 22). It also provides evidence that a subclinical abnormality associated with N_2_O, combined with rapid consumption of N_2_O, may have contributed to the onset of symptoms.

In addition to the factors associated with a worse clinical presentation, the demographics of these patients also warranted consideration. Although these characteristics were not related to clinical severity in our study, they may indicate which group of people is more likely to consume N_2_O. Therefore, clinicians would be alerted to inquire about N_2_O consumption if they encounter neuropathy symptoms in a patient. According to our patients, most were in their early twenties and unemployed. Past neuropsychological history was common, for example, depression, bipolar disorder, and sleep disorders. More than half of them regularly and heavily consumed cigarettes, alcohol, or narcotics, almost doubling or tripling the rates in the general same-aged population in China (23, 24). Similar to our cohort, high psychiatric comorbidity, substance use, and unemployment rates have been reported in other N_2_O cohorts (21, 25–27). Social factors could also have an impact on mental health. For example, the recent COVID-19 pandemic has also been shown to lead to an increase in drug and alcohol abuse, including the consumption of N_2_O (28). In addition, 30% of our patients initially concealed their use of N_2_O due to the aforementioned psychosocial factors and the cultural perception in China that the consumption of psychoactive substances is unacceptable. The concealment of substance abuse could be complicated if patients present with atypical symptoms upon admission, such as neuropsychiatric symptoms. There was an unemployed 19-year-old patient who initially presented with delusions, hallucinations, and depression in our cohort. During hospitalization in the psychiatric ward, he developed an unsteady gait and frequent falling. The consultant neurologist diagnosed him with Guillain–Barré syndrome because he was unable to obtain a positive N_2_O consumption history. After discharge, the patient re-used N_2_O and was readmitted to the hospital; it was only then that a positive history of consumption was obtained, and the cause of the initial psychiatric symptoms was identified. Therefore, when encountering a patient with neuropathy with the aforementioned demographic traits, physicians should also focus on collecting a detailed substance use history, including consumption of N_2_O.

Neuroimaging and other medical examinations helped make the diagnosis, but they lacked sensitivity and specificity. Abnormal spinal MRI was found in 37.5% of our patients, lower than other N_2_O cohorts (47–100%), but the cervical spinal cord was consistent to be found as the most frequently involved segment among published literature (6, 10–12, 19, 21, 29). Results from other large cohorts have shown the lesions on MRI often extend 4–6 spinal segments and are presented as inverted “V,” “Triangle,” or “oval” shapes in the posterior column of the spinal cord (6, 10). Compared with other SCDs, Gao et al. have found that N_2_O-related SCDs had higher rates of spinal MRI abnormality, wider spinal lesions on sagittal MRI, fewer involved spinal segments, and higher incidence of inverted “V” sign (10). Since there are substantial differences in percentages of abnormalities between cohorts and there are no specific MRI findings in N_2_O cases, the findings have limited significance. On the EMGs, lower limb motor axonal dysfunction was predominant among our cases, similar to findings from the initial report in 1978 (3, 4) and a detailed EMG study of two N_2_O cases (30). However, the EMG findings reported from published studies were heterogeneous, with most patients reported to have both axonal and demyelinating sensorimotor neuropathies (11, 19, 31). N_2_O may interfere with the VitB12-dependent enzymes (methionine synthase and MMCoA mutase) and cause a lack of myeline methylation, axonal regeneration disruption, and myelin integrity destruction, according to published studies on the mechanisms of N_2_O neural injury (21). Thus, both axonal and demyelinating neuropathies were detectable on EMG. The inconsistent clinical findings among studies might be due to the size of included patients and the different ways of EMG analysis. The motor neuropathy observed in N_2_O cases may be attributed to factors other than VitB12 deficiency, as VitB12 is known to affect the sensory nerves predominantly (32, 33). For example, N_2_O-induced repeated hypoxia and destruction of cytokine balance could also participate in the pathogenesis processes (6, 34).

## Limitations

First, because the participants were hospitalized cases from a tertiary medical center, the clinical presentation was likely to be more severe than in non-hospitalized cases. Second, the cohort had a low natural follow-up rate, multiple psychosocial factors, and a tendency to conceal N_2_O use. These factors made it challenging to conduct thorough follow-ups. As a result, we were unable to provide detailed recovery status information and analyze follow-up data for these individuals.

## Conclusion

Recreational N_2_O use could cause severe neuropathies. Changes in the RBC index could be an early indicator of a more severe clinical presentation. High rates of psychiatric comorbidity and complex psychosocial factors make it more difficult to obtain a patient's medical history and to manage the patient. Clinicians should collect N_2_O consumption histories from patients with neuropathy with specific psychosocial characteristics. An emphasis should also be placed on population education and control measures regarding access to N_2_O.

## Data availability statement

The raw data supporting the conclusions of this article will be made available by the authors, without undue reservation.

## Ethics statement

The studies involving human participants were reviewed and approved by the Ethics Committee of West China Hospital of Sichuan University. The patients/participants or their legal guardians provided their written informed consent to participate in this study.

## Author contributions

HG, QC, and DZ were involved in conceptualization. HG, RW, YZ, LQ, and HC helped in data collection and analysis. HG and RW wrote the original draft of the manuscript. QC reviewed and edited the draft. All authors contributed to the article and approved the submitted version.
