# Supplementary material for: Nitrous oxide-induced neurotoxicity: Clinical characteristics and impacts on overall neurological impairments
Source: Front Neurol. 2023 Feb 23;14:1132542. doi: 10.3389/fneur.2023.1132542 (PMC9995698; doi:10.3389/fneur.2023.1132542)
Supplement: Supplementary file 1 [file Table_1.DOCX]

**TABLE S1. Correlation between the Functional Disability Rating Score (FDRS) sum score and clinical characteristics**

| **Clinical characteristics** | **FDRS sum score** |
| --- | --- |
| **Demographics** | |
| Gender | 0.152 (*p* = 0.522) |
| Age | -0.384 (*p* = 0.095) |
| Unemployed | 0.018 (*p* = 0.941) |
| Positive past neuropsychological history | -0.315 (*p* = 0.177) |
| Positive substance consumption history | 0.044 (*p* = 0.853) |
| **N_2_O consumption** | |
| N_2_O exposure time | -0.212 (*p* = 0.384) |
| Recent increased use of N_2_O | 0.394 (*p* = 0.205) |
| Concealment of the use of N_2_O | 0.269 (*p* = 0.252) |
| **Records of hospitalization and follow-up** | |
| Onset-to-admission times | -0.409 (*p* = 0.073) |
| Admitted from Emergency Room | 0.477 (*p* = 0.033) |
| Length of hospital stay | 0.140 (*p* = 0.556) |
| Natural follow-up rate | 0.062 (*p* = 0.797) |
| **Spine MRI** | 0.000 (*p* = 1.000) |
| Cervical | 0.083 (*p* = 0.769) |
| Thoracic | -0.117 (*p* = 0.677) |
| Lumbar | / |
| **First blood lab results without previous VitB12 supplements** | |
| Decreased Hb | 0.817 (*p* = 0.004) |
| Increased MCV | 0.273 (*p* = 0.445) |
| Decreased RBC count | 0.817 (*p* = 0.004) |
| VitB12 | 0.617 (*p* = 0.140) |
| VitB12 deficiency | -0.302 (*p* = 0.510) |
| Increased Hcy | 0.000 (*p* = 1.000) |
| Decreased intrinsic factor antibody | 0.000 (*p* = 1.000) |
| **First blood lab results with previous VitB12 supplements** | |
| Decreased Hb | -0.664 (*p* = 0.150) |
| Increased MCV | 0.840 (*p* = 0.036) |
| Decreased RBC count | -0.210 (*p* = 0.690) |
| VitB12 | -0.493 (*p* = 0.321) |
| VitB12 deficiency | / |
| Increased Hcy | 0.258 (*p* = 0.742) |
| Decreased intrinsic factor antibody | 0.725 (*p* = 0.165) |

Abbreviations: Hb, hemoglobin; MCV, mean corpuscular volume; RBC, red blood cell; Hcy, homocysteine; MRI, Magnetic Resonance Imaging; IQR, interquartile range; VitB12, vitamin 12.
